# CD160 and PD-1 Co-Expression on HIV-Specific CD8 T Cells Defines a Subset with Advanced Dysfunction

**DOI:** 10.1371/journal.ppat.1002840

**Published:** 2012-08-16

**Authors:** Yoav Peretz, Zhong He, Yu Shi, Bader Yassine-Diab, Jean-Philippe Goulet, Rebeka Bordi, Ali Filali-Mouhim, Jean-Baptiste Loubert, Mohamed El-Far, Franck P. Dupuy, Mohamed Rachid Boulassel, Cécile Tremblay, Jean-Pierre Routy, Nicole Bernard, Robert Balderas, Elias K. Haddad, Rafick-Pierre Sékaly

**Affiliations:** 1 Caprion/ImmuneCarta Services, Montreal, Quebec, Canada; 2 Centre de Recherche du Centre hospitalier de l'Université de Montréal (CRCHUM), Hôpital St-Luc, Montreal, Quebec, Canada; 3 Laboratoire d'Immunologie, Département de Microbiologie et d'Immunologie, Université de Montreal, Montreal, Quebec, Canada; 4 Vaccine & Gene Therapy Institute Florida, Port St. Lucie, Florida, United States of America; 5 Immunodeficiency Service and Division of Hematology, Royal Victoria Hospital, McGill University Health Centre, McGill University, Montreal, Quebec, Canada; 6 Department of Experimental Medicine, McGill University, Montreal, Quebec, Canada; 7 BD Biosciences, San Diego, California, United States of America; 8 Department of Microbiology and Immunology, McGill University, Montreal, Quebec, Canada; 9 Institut National de la Santé et de la Recherche Médicale U743, CRCHUM, Université de Montreal, Montreal, Quebec, Canada; Emory University, United States of America

## Abstract

Chronic viral infections lead to persistent CD8 T cell activation and functional exhaustion. Expression of programmed cell death-1 (PD-1) has been associated to CD8 T cell dysfunction in HIV infection. Herein we report that another negative regulator of T cell activation, CD160, was also upregulated on HIV-specific CD8 T lymphocytes mostly during the chronic phase of infection. CD8 T cells that expressed CD160 or PD-1 were still functional whereas co-expression of CD160 and PD-1 on CD8 T cells defined a novel subset with all the characteristics of functionally exhausted T cells. Blocking the interaction of CD160 with HVEM, its natural ligand, increased HIV-specific CD8 T cell proliferation and cytokine production. Transcriptional profiling showed that CD160^−^PD-1^+^CD8 T cells encompassed a subset of CD8^+^ T cells with activated transcriptional programs, while CD160^+^PD-1^+^ T cells encompassed primarily CD8^+^ T cells with an exhausted phenotype. The transcriptional profile of CD160^+^PD-1^+^ T cells showed the downregulation of the NFκB transcriptional node and the upregulation of several inhibitors of T cell survival and function. Overall, we show that CD160 and PD-1 expressing subsets allow differentiating between activated and exhausted CD8 T cells further reinforcing the notion that restoration of function will require multipronged approaches that target several negative regulators.

## Author Summary

HIV infection is widely known to cause generalized immune activation and immune exhaustion ultimately leading to HIV disease progression. Several studies have suggested over the years that the accumulation of inhibitory signalling proteins on the surface of responding cells is linked to immune exhaustion in HIV. It has become paramount to distinguish functionally exhausted CD8 T cells from activated HIV-specific CD8 T cells because both cell types have different fates. Using specific cell surface markers, we were able to identify these different cell types and show that HIV-infected patients accumulate dysfunctional CD8 T cells over time. Importantly, we show that this dysfunction is reversible.

## Introduction

Mounting evidence supports the notion that CD8 T cells contribute to the control of HIV viral replication [1]. The emergence in early infection of viral variants bearing escape mutations within sequences targeted by HIV-specific T lymphocytes is consistent with CD8 T cells exerting selective antiviral pressure [2]–[3]. However, HIV viral replication outpaces the adaptive immune response leading to the establishment of chronic infection partly because CD8 T cells are progressively deleted [4] and/or become dysfunctional [5]–[6].

Functionally exhausted T cells were originally described in a murine model of acute and chronic lymphocytic choriomeningitis virus (LCMV) infection whereby virus-specific CD8 T cells persist but lack effector function [7]. Exhausted CD4 and CD8 T cells have since been described in cancer [8] and chronic viral infections such as SIV [9], HIV [10]–[12], Hepatitis C (HCV) [13]–[14] and Hepatitis B (HBV) [15]. Functional impairment of antigen-specific responses has been shown to occur in a stepwise and hierarchical manner with proliferation, IL-2 and TNFαproduction being the first functions lost followed by IFNγ [16]–[18]; eventually cells die by apoptosis [19]. Of note, in chronic LCMV infection inhibitory receptors such as PD-1, LAG-3, CD160, CTLA-4, 2B4/CD244, GP49 and PirB are all upregulated on exhausted LCMV-specific CD8 T cells compared to functional effector or memory cells [20]–[21]. Blocking the interaction of PD-1 and LAG-3 with their natural ligands restored virus specific T cell proliferation and effector cytokine production (IFNγ, TNFα and CD107a) in this mouse model. However, functional restoration was only partial suggesting the involvement and cooperation of several negative regulatory pathways [20] in the programming of T cell exhaustion.

Several mechanisms that lead to antigen-specific CD8 T cell dysfunction in HIV infection have also been described; they include the lack of CD4 help [22] as well as the upregulation on HIV-specific and total T cells of several negative regulators of T cell activation including PD-1, CTLA-4, CD160, 2B4 and Tim-3. PD-1 [23]–[25], CTLA-4 [26] and Tim-3 [27]–[28] have also been associated to HCV and HIV-specific CD4 and CD8 T cell dysfunction as the expression levels of these molecules correlated positively with plasma viral load and negatively with absolute CD4 T cell counts while there expression declined in subjects treated with highly active antiretroviral therapy (HAART) [23], [26]–[27].More recently, studies have shown in cancer [29] and HIV [30]–[31] that the co-expression of several immune inhibitory molecules on antigen-specific CD8 T cells leads to a more severe dysfunction. Of note, expression of these molecules is a by-product of T cell activation as they play an important role in T cell homeostasis. Therefore, the mechanisms that determine the functions of these molecules in T cell activation and in functional T cell exhaustion have been difficult to decipher, as most of these molecules are also upregulated upon T cell activation.

We therefore analyzed the expression and function of CD160 and PD-1 on CMV and HIV-specific CD8 T cells during different stages of infection and identified 4 functionally distinct subsets of CD8 T cells (CD160^−^PD-1^−^, CD160^−^PD-1^+^, CD160^+^PD-1^−^, CD160^+^PD-1^+^). Our data identified a unique CD160^+^PD-1^+^ subset at an advanced stage of exhaustion mostly found during chronic HIV infection (CHI).

## Results

### CD160 Expression Is Preferentially Upregulated on HIV-Specific CD8 T Cells

Microarray analysis of sorted CMV and HIV-specific CD8 T cells in 27 HIV-infected subjects, showed a significant increase (1.7 fold and p<0.05) in levels of expression of CD160 mRNA in HIV-specific CD8 T cells when compared to CMV-specific CD8 T cells (Figure S1). To confirm the results generated by microarray, we measured the levels of CD160 expression on CMV and HIV-specific CD8 T cells as well as total CD8 T cells isolated from 7 HIV-uninfected individuals and 38 HIV-infected subjects divided into 4 groups: acute HIV infection (AHI; n = 7), chronic/progressing infection (CHI; n = 9), successfully treated and aviremic individuals (ST; n = 12) and Elite controllers (ECs; n = 10) (Table 1, 2). HIV and CMV-specific MHC class I tetramers were used to identify these cells. We also measured the expression of PD-1 on cells from individuals in these different groups as this molecule has also been shown to be upregulated on HIV-specific T cells as well as total CD8 T cells in HIV infection.

**Table 1:** Clinical characteristics of study population.

Patient ID	Sex	Duration of infection (months)	CCR5	A	B	C	CD4 cells/mm^3^	CD8 cells/mm^3^	LOG VL (HIV copies/ml)
**Elite Controllers (EC)**									
LPN001	M	116.7	WT	2,2	27,40	2,15	860	1148	1.70
LTNP HTM 006	F	164.8		2,74	27,49	2,7	692	627	1.70
LTNP006	F	132.2	HTZ	2,68	44,57	5,6	991	921	1.70
LTNP-CQL008	M	137.0	WT	2	27,40	2,15	1040	1094	1.70
LTNP-CQL005	M	240.6	WT	1,32	27,57	1,18	1080	1333	1.70
LTNP-HDM008	F	168.8		2,3	7,18	7	800	1030	1.70
LTNP-HTM001	F	141.7	WT	3,11	15,35	3,12	737	303	1.70
LTNP003	M	189.8	WT	2,68	14,15	4,8	445	1514	1.70
LTNP007	M	131.5	WT	1,3	8,14	7,8	720	631	1.70
LTNP-015	F	166.3	HTZ	2,26	7,38	7,12	671	626	1.70
**Successfully treated (ST)**									
ST002		86.1		2,31	14,35	4,8	599	923	1.70
ST004		157.6		2,30	18,40	3,5	602	767	1.70
HTM332	F	34.4	WT	2,33	53,58	3,4	356	629	1.70
ST015		138.7		2,3	8,14	7,8	552	715	1.70
ST016		242.4		2,68	15,44	1,5	671	1120	1.70
HTM305	M	73.0	WT	11,24	8,35	4,7	222	838	1.70
HTM308	M	73.7	HTZ	2,11	8,52	7,12	691	631	1.70
HTM318	M	61.0		3,24	18,35	4,12	915	750	1.70
ST007		86.2		2,24	35,51	4,12	563	613	1.70
HTM322	M	94.0	WT	2,68	14,44	5,8	235	399	1.70
HTM326	M	85.9	WT	2,24	18,44	5,12	883	333	1.70
HTM338	M	18.4	WT	3,68	7,14	7,8	443	332	1.70
**Acute**									
HDM013	F	1.7	HTZ	2,32	18,35	4,7	640	1990	5.45
HTM365	M	1.6	WT	2,29	18,44	5,7	257	1007	6.59
DRPI041	M	1.9	HTZ	2	7,8	5,7	475	640	4.75
DRPI049	M	2.6	WT	1,2	8,51	2,7	355	1012	4.47
HDM011	M	1.7	HTZ	1	8	7	594	692	5.23
DRPI040	M	2.5	WT	2,29	40,51	1,3	555	1146	4.13
NYU008	M	2.9		3,26	8,27	1,7	267	728	5.08
**Chronics**									
HDM003	M	16.5	WT	2,3	7,15	7	494	1055	4.20
ACT42117	M	7.9		2,11	7,8	7	704	1081	4.84
DRPI040	M	11.7	WT	2,29	40,51	1,3	345	511	4.86
DRPI025	M	10.3	WT	32,34	8,15	3,7	475	410	5.39
DRPI026	M	51.5	WT	3,29	7,44	7,16	450	898	5.24
DRPI037	M	10.7	WT	3,68	7,14	7,8	858	677	4.00
IDR192	M	57.1	WT	23,30	7,15	3,7	368	1320	4.54
IDR194	M	34.8	WT	2,24	18,44	5,12	383	354	4.32
DRPI041	M	33.3	HTZ	2	7,8	5,7	296	717	5.24
**HIV uninfected**									
L604									
L584									
L606									
L145									
L577									
L614									
L761									

**Table 2:** Summary of clinical characteristics.

	*Median Time from infection (months)*	*n*	*Median CD4 (cells/mm^3^)*	*Median CD8 (cells/mm^3^)*	*Median Log VL (copies/ml of plasma)*
***Elite controllers (EC)***	153.2 (116.7–240.6)	10	769 (445–1080)	976 (303–1514)	<1.7
***Successfully Treated (ST)***	86.0 (18.4–242.4)	12	581 (222–915)	631 (332–1120)	<1.7
***Acute (AHI)***	1.9 (1.6–2.9)	7	475 (257–640)	1007 (692–1990)	5.1 (4.13–6.59)
***Chronics (CHI)***	16.5 (6.4–57.1)	9	450 (296–858)	717 (354–1320)	4.8 (4.0–5.39)
***HIV uninfected***		7			

Results illustrated in Figure S2A and S2B showed that significantly higher levels of CD160, as monitored by Mean Fluorescence Intensity (MFI), were found on HIV-specific CD8 T cells when compared to CMV-specific CD8 T cells in CHI subjects (CMV: MFI = 1817; HIV: MFI = 4636; *P* = 0.0001) and ECs (CMV: MFI = 2428; HIV: MFI = 3610; *P* = 0.009) (Figure S2B; left panel). As well, the frequency of HIV-specific CD160^+^ T cells was significantly higher than that of CD160^+^ CMV-specific T cells in CHI (CMV = 42.5%; HIV = 70.7%; *P* = 0.0001) and ECs (CMV = 56.1%; HIV = 74.1%; *P* = 0.009) (Figure S2B; right panel). The increased expression of CD160 on HIV-specific CD8 T cells was observed only during the chronic phases of HIV infection. Importantly, HIV and CMV-specific CD8 T cells expressed similar levels of CD160 in the acute phase of infection. The MFI and frequencies of CD160 on antigen-specific CD8 T cells did not differ between ST and ECs. As shown previously [23]–[24], the frequency of PD-1^+^ HIV-specific T cells as well as the levels of expression of this molecule were greater on HIV than CMV-specific CD8 T cells during CHI (Figure S2C,D).The MFI and frequency of PD-1 on HIV-specific T cells were significantly lower in ECs (54.2%; MFI = 3479) compared to AHI (69.8%, *P* = 0.0004; MFI = 5742, *P*<0.0042),CHI (86.0%, *P*<0.0001; MFI = 8377, *P* = 0.0002) and ST patients (76.1%, *P* = 0.03; MFI = 5097, *P* = 0.006).

Total CD8 T cells in CHI subjects and ECs also included a significantly higher proportion of cells expressing high levels of CD160 compared to levels observed in uninfected controls (MFI = 1251 and 24.4% in uninfected individuals; MFI = 1340 and25.3% in AHI; MFI = 1983and 35.4% in CHI; MFI = 1788 and 36.0% in ECs) (Figure S3). As shown for antigen-specific responses, total CD8 T cells upregulated CD160 expression only during the chronic stage of infection. CD160 expression was restricted to terminally differentiated (CD45RA^+^CD27^−^CCR7^−^) and memory CD8 T cells (CD45RA^−^CD27^+/−^CCR7^+/−^) whereas naïve CD8 T cells (CD45RA^+^CD27^+^CCR7^+^) did not express this protein (Figure S4). Of note, while PD-1 levels were upregulated on HIV-specific and total CD8 T cells in AHI and CHI compared to uninfected subjects, elevated CD160 levels were limited to CHI. Our results highlight a difference in the timing of expression of these molecules during the natural history of HIV infection.

### Co-Expression of CD160 and PD-1 on CD8 T Cells Is a Marker of Disease Progression

**Figure 1:**
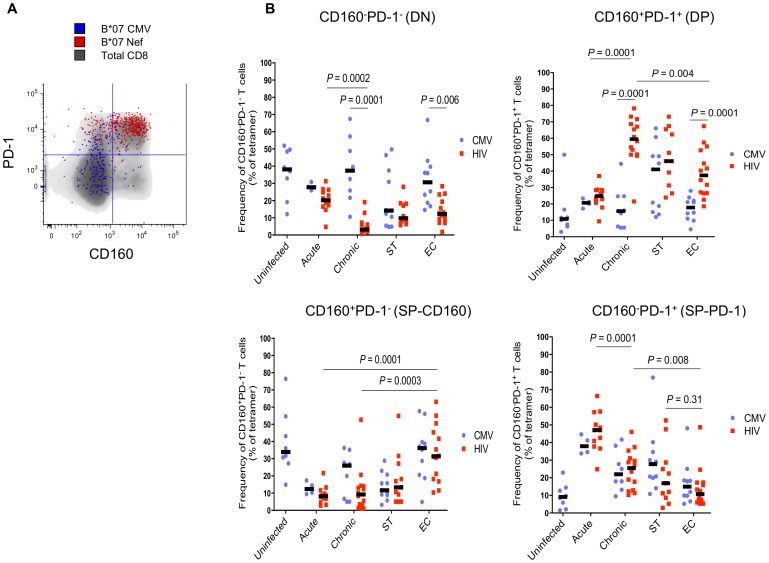
CD160 and PD-1 expression defines 4 distinct subsets of HIV-specific CD8 T cells. (A) Representative flow cytometry plot of CD160 and PD-1 co-expression on total, CMV and HIV-specific CD8 T cells. (B) Scatter plots represent the median frequency of 4 distinct subsets of CD8 T cells based on CD160 and PD-1 expression (CD160^−^PD-1^−^; CD160^−^PD-1^+^; CD160^+^PD-1^−^; CD160^+^PD-1^+^) from 7 HIV-1 uninfected and 38 HIV-infected subjects separated into four groups: 7 during acute/early infection (AHI), 9 chronic progressors (CHI), 12 successfully treated(ST) and 10 Elite controllers (ECs). PBMCs were labelled with fluorochrome conjugated αCD3, αCD8, αPD-1,αCD160 and HLA class I-matched tetramers (see materials and methods). Dying cells were eliminated with an amine-reactive viability dye. Blue and red dots represent CMV and HIV-specific CD8 T cells, respectively. *P*-values were determined by Mann Whitney and unpaired *t* tests.

We measured the co-expression of CD160 and PD-1 on HIV and CMV-specific CD8 T cells in the groups of subjects described above. Four distinct cell subsets were identified: CD160^−^PD-1^−^ (DN), CD160^−^PD-1^+^ (SP-PD-1), CD160^+^PD-1^−^ (SP-CD160) and CD160^+^PD-1^+^ (DP) (Figure 1A, Figure S5A,B). Frequencies of DN and DP subsets within HIV-specific T cells showed significant differences when comparing cross-sectionally HIV-infected subjects at different stages of disease (AHI vs CHI). The percentages of HIV-specific DN cells were highest in AHI (20.2% range, 4.7–31.3) and lowest in CHI (3.2% range, 0.7–19.1) (*P* = 0.0002)(Figure 1B; upper left panel). In contrast, frequencies of HIV-specific DP cells were highest in CHI (59.5% range, 21.5–78.2) and their numbers were the lowest in AHI (24.8% range, 9.4–37.0) (*P* = 0.0001)(Figure 1B; upper right panel). In this cross-sectional analysis, the frequencies of DN and DP subsets within CMV-specific T cells did not vary between acute and chronic infection (Figure 1B).Elite controllers also showed high frequencies of HIV-specific T cells with a DP phenotype; however frequencies were significantly lower compared to CHI (*P* = 0.004) (Figure 1B; upper right panel). As for the two SP subsets, their frequencies significantly shifted when comparing individuals at different stages of disease. The frequency of SP-PD-1 HIV-specific CD8 T cells was highest during AHI (47.1%) and significantly decreased in CHI (25.5%; *P* = 0.0001) and ECs (14.0%, *P* = 0.008) while the frequencies of SP-CD160 CD8 T cells were higher in ECs compared to other study groups (Figure 1B; lower panels). The frequencies of SP-PD-1 and DP subsets from ST subjects did not significantly differ from those observed in CHI and EC. Interestingly, the frequency of SP-PD-1 HIV-specific CD8 T cells did not differ from that of SP-PD-1 T cells recognizing CMV epitopes at all stages of HIV infection (Figure 1B; lower right panel). These results indicated that HIV-specific CD8 T cells expressing PD-1 encompass multiple subsets. Importantly, the phenotype of these cells was shown to be different when comparing CD8 T cells in acute and chronic HIV infection. Moreover, the level of PD-1 expression was highest on CD160^+^CD8 T cells(Figure S5C), further reinforcing the notion that DP cells are at an advanced stage of exhaustion and raising the possibility that SP-PD-1 cells encompass recently activated T cells [32]–[33]

The lower frequencies of HIV-specific DP CD8 T cells in ECs compared to CHI subjects (Figure 1) suggested the involvement of CD160 and PD-1 co-expression in antigen-specific T cell dysfunction as previously shown in mice models of chronic viral infection [20]–[21] and suggested recently in HIV-infected subjects [27], [30].

### The Frequency of CD160 and PD-1 Co-Expression Increases with Disease Progression

**Figure 2:**
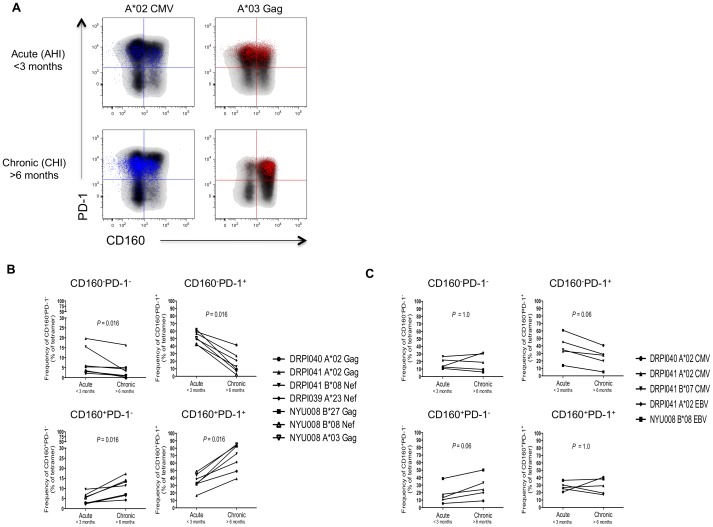
The frequency of HIV-specific CD8 T cells with a CD160^+^PD-1^+^ phenotype increases with disease progression. (A) Representative flow cytometry plots of CMV and HIV-specific CD8 T cells (A*02 CMV and A*03 Gag) detected during AHI (<3 months) and CHI (>6 months) within the same individual. Frequencies of 4 distinct CD8 T cell subsets expressing CD160 and/or PD-1 were measured at both time points. Figures represent the frequency of CD160 and/or PD-1 expressing subsets over time for (B) HIV and (C) CMV/EBV-specific responses in 4 HIV-infected subjects. *P*-values were determined by the Wilcoxon matched pairs test.

A longitudinal analysis was performed to confirm the dynamic evolution of these phenotypes during different disease stages. We assessed the frequencies of CMV, EBV and HIV-specific CD8 T cells expressing CD160 and/or PD-1 in 5 HIV-infected subjects during AHI (<3 months on infection) and CHI (>6 months of infection) (Figure 2A). The frequency of HIV-specific PD-1 expressing CD8 T cells (SP-PD-1 and DP) remained stable over time(Figure S6). However, when we assessed the two subsets of PD-1^+^ cells, we observed a significant decrease in the frequencies of SP-PD-1 HIV-specific CD8 T cells (*P* = 0.016) over the course of infection whereas the frequencies of DP CD8 T cells significantly increased (*P* = 0.016) from AHI to CHI (Figure 2B; right panels) confirming results obtained in the cross-sectional analysis. In contrast, the proportion of CD160^+^ HIV-specific CD8 T cells (SP-CD160 and DP) increased with disease progression (Figure S6). Percentages of DN and SP-CD160 HIV-specific CD8 T cells increased from AHI to CHI (*P* = 0.016) (Figure 2B; left panels). The distribution of CD160 and PD-1 subsets on cells specific for CMV and EBV epitopes did not significantly change from AHI to CHI (Figure 2C). Taken together, these results showed a dynamic evolution of the frequency and distribution of CD160 and/or PD-1 expression on HIV-specific CD8 T cells during infection. Both cross-sectional and longitudinal results confirmed that the distribution of CD160 and PD-1 expressing subsets was predominantly SP-PD-1 in AHI and DP in chronic HIV infection. Our results strongly indicated that the simultaneous expression of CD160 and PD-1 could constitute a marker of T cell exhaustion and disease progression. A recent study published by Youngblood et *al.* [34] reinforced this observation by showing that persistent TCR signalling results in sustained PD-1 expression by maintaining PD-1regulatory regions accessible to transcription factors.

### CD160 Expression Characterizes HIV-Specific T Cell Dysfunction

**Figure 3:**
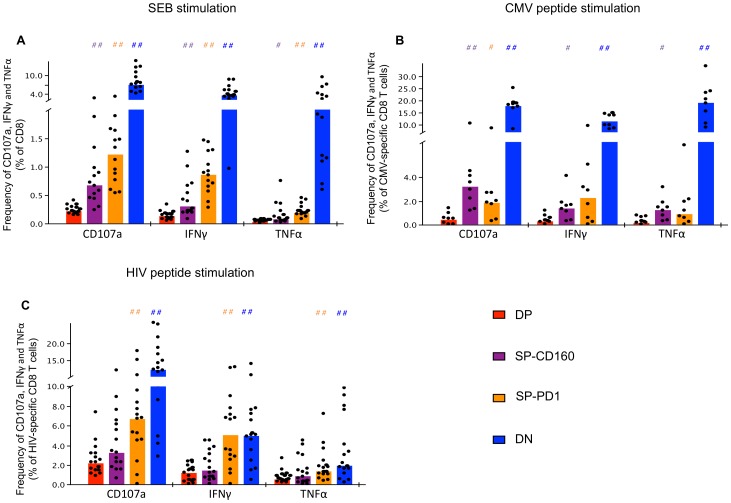
Co-expression of CD160 and PD-1 identifies a fully exhausted antigen-specific CD8 T cell. PBMCs were isolated from HIV viremic individuals (n = 14) and stimulated with (A) SEB, (B) CMV and (C) HIV-1 peptides and analyzed by multiparametric flow cytometry for CD160, PD-1, IFNγ, TNFα and CD107a expression. HIV and CMV-specific CD8 T cells were identified using PE-conjugated tetramers. Dying cells were eliminated with an amine-reactive viability dye. # symbols represent statistically significant comparisons with the DP subset. *P*-values were determined by the Wilcoxon matched pairs test. ##represents a *P* value<0.005 and # represents a *P* value<0.05.

We next assessed the effector function of these different subsets by intracellular cytokine staining (ICS) for IFNγ and TNFαsecretion and measured the expression of CD107a following stimulation with SEB, CMVpp65 and HIV peptides in viremic HIV-infected subjects. Figure 3A depicts the percentage of cytokine secreting cells within the total CD8 T cell population upon SEB stimulation. Frequencies of CD8 T cells producing TNFα, IFNγ or upregulating CD107a were significantly higher in DN, SP-PD-1 and SP-CD160 compared to frequencies observed in the DP subset (*P*<0.05 and *P*<0.005, #or ## represents significant change in cytokine production compared to DP). Furthermore cells with a DN phenotype had higher frequencies of functional CD8 T cells when compared to SP-CD160 and SP-PD-1 following stimulation with SEB. In summary, the expression of either CD160 or PD-1 on CD8 T cells identified a T cell subset having lower levels of effector function (IFNγ, TNFα) or degranulation (CD107a). Importantly, our results indicated that cells expressing both CD160 and PD-1 were more dysfunctional than cells expressing either one of these two molecules.

The same hierarchy of functionality was observed when comparing the four different subsets of T cells following stimulation with individual CMV peptides (Figure 3B). As observed above, DN cells showed the highest frequencies of cells that produced TNFα (19.2%), IFNγ (11.5%), and upregulated CD107a (17.6%) when compared to DP cells (TNFα0.4%, IFNγ0.5%, CD107a 0.5%). SP-PD-1 (TNFα = 1.7%, IFNγ = 2.9%, CD107a = 2.5%)and SP-CD160 (TNFα = 1.3%, IFNγ = 1.5%, CD107a = 3.8%) also showed lower frequencies of cytokine producing cells than DN and importantly higher frequencies than DP cells.

Triggering of HIV-specific cells yielded similar results to SEB and CMV stimulated T cells. As noted above, DP (TNFα: 0.8%, IFNγ:1.2%, CD107a:2.6%) and DN (TNFα:3.5%, IFNγ:5.4%, CD107a:13.4%) cells showed the most significant difference in the frequency of cytokine producing cells with a downregulation of TNFα, IFNγand CD107a expression (Figure 3C). Following stimulation with HIV peptides, the SP-PD-1 (TNFα:1.9%, IFNγ:5.4%, CD107a:7.0%) subset included significantly higher frequencies of IFNγ, TNFα secreting cells as well as CD107a^+^ degranulating cells as compared with the DP subset (Figure 3C; ##represents significant change in cytokine production compared to DP). As observed for SEB and CMV peptide stimulated CD8 T cells, DP CD8 T cells were consistently the least functional subset.

Together these results showed that antigen-specific CD8 T cells with a DP phenotype were less functional than SP-PD-1 expressing CD8 T cells as shown by their lower responses to all three T cell activation signals. These results provide evidence that co-expression of CD160 and PD-1identified dysfunctional CD8 T cells. This degree of dysfunctionality progressively increases with the co-expression of additional immune inhibitory markers on CMV and HIV-specific T cells.

### Blocking the Interaction between CD160 and HVEM Enhances and Rescues CMV and HIV-specific CD8 T Cell Proliferation and Cytokine Production

**Figure 4:**
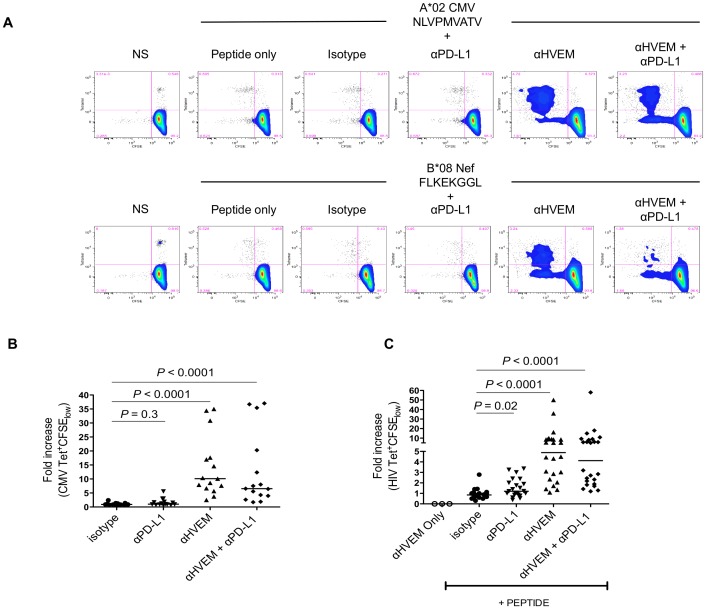
Blocking the interaction between CD160 and HVEM enhances CMV and HIV-specific CD8 T cell proliferation and cytokine production. HIV-infected individuals(n = 11) were stimulated with HLA-restricted CMV and HIV peptides in the presence of blocking antibodies to HVEM and/or PD-L1. Dying cells were eliminated with an amine-reactive viability dye and PBMCs were stained at day 6 with HLA class I matched-tetramers and mAbs to CD3 and CD8. (A) Representative flow cytometry plots of an HIV-infected patient stimulated for 6 days with a CMV and HIV peptide in the presence of isotype, αPD-L1 and/or αHVEM blocking antibodies. Scatter plots represent the median fold increase in (B) CMV and (C) HIV-specific proliferation (Tetramer^+^/CFSE_low_) compared to the isotype control. Each dot represents a CMV or HIV tetramer-specific response. *P*-values were determined by the Wilcoxon matched pairs test.

HVEM is the natural ligand of CD160 [35]. We therefore performed experiments aimed at determining whether interfering with CD160 engagement by HVEM allowed dysfunctional T cells to recover their effector T cell function. PBMCs were stimulated with HLA-restricted CMV and HIV optimal peptides in a 6-day CFSE assay in the presence or absence of αHVEM together with or without αPD-L1 blocking antibodies (Figure 4A). We measured the expression of HVEM on monocytes, mDCs and pDCs and observed a significant upregulation of HVEM surface expression on monocytes and pDCs from CHI individuals compared to healthy controls. Similar findings were observed when measuring the expression of PD-L1 on monocytes (*P*<0.05) (Figure S7). Our results showed that blocking CD160 interaction with HVEM significantly enhanced CMV and HIV-specific CD8 T cell proliferation (Figure 4B,C). We observed a median fold increase of 10.1 (*P*<0.0001) and 4.9 (*P*<0.0001) in CMV and HIV-specific CD8 T cell proliferation respectively, compared to isotype controls. Blocking the PD-1/PD-L1 pathway led to a statistically significant enhancement of HIV-specific CD8 T cell proliferation by a factor of 1.2 (*P* = 0.02); hence CD160/HVEM blockade was more potent than PD-1/PD-L1 blockade at restoring HIV-specific CD8 T cell proliferation. PBMCs cultured with both blocking antibodies (αPD-L1 and αHVEM) also significantly enhanced T cell proliferation however, the effect was not synergistic compared to using αHVEM alone (*P*<0.0001) (Figure 4B,C).We analyzed the coexpression of BTLA and CD160 on HIV-specific CD8 T cells during chronic HIV infection and found that the frequency of CD160 (35.5%) was significantly greater than BTLA (3.9%; *P*<0.0001) suggesting that using αHVEM preferentially disrupts the CD160/HVEM axis (Figure S8). Controls showed that the αHVEM blocking antibody did not induce T cell activation in the absence of the T cell cognate peptide.

Supernatants harvested following the 6-day CFSE assay were used to assess cytokine production in the presence or absence of PD-1 and or CD160 engagement by their respective ligands. We found that levels of IFNγ, IL-4 and IL-10 were significantly increased compared to isotype controls in conditions where αHVEM was added to the T cell cultures (IFNγ, *P* = 0.001; IL-4, *P* = 0.03; IL-10, *P* = 0.003) (Figure S9). Although TNFα production was increased in conditions where αHVEM was present, the levels were not statistically different compared with the isotype control (TNFα, *P* = 0.054). The levels of IL-2 production did not significantly increase upon antigen-specific stimulation in the presence of HVEM blockade most likely due to the consumption of this cytokine by proliferating T cells. These results confirmed that CD160 was implicated in HIV-specific CD8 T cell exhaustion. Blocking its interaction with HVEM restored the proliferation and cytokine production of antigen-specific CD8 T cells.

### CD160^+^PD-1^+^CD8 T Cells Represent a Distinct CD8 T Cell Subset with a Unique Transcriptional Profile

**Figure 5:**
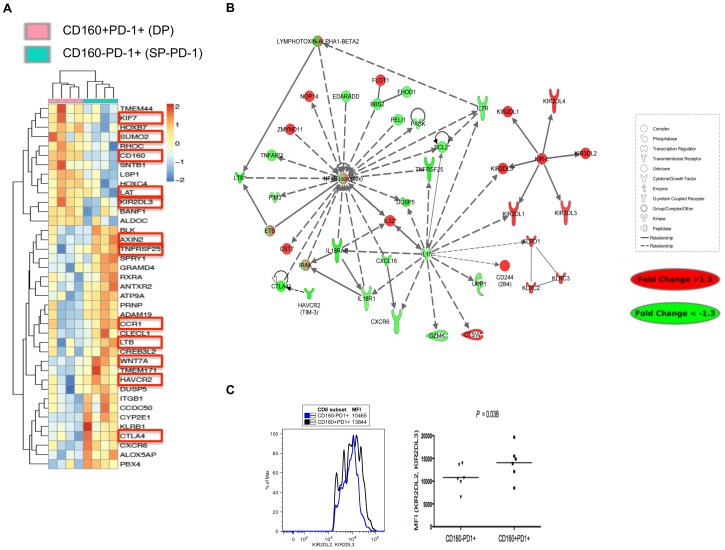
CD160^+^PD-1^+^CD8 T cells represent a distinct subset with a unique transcriptional profile. PBMCs were stained with 7AAD (cell viability dye), αCD3, αCD8, αPD-1, αCD160 and total CD8 T cells were sorted using a FACS ARIA based on CD160 and PD-1 expression. (A) Heatmap illustrating the differentially expressed genes (39 genes; p<0.05) between DP and SP-PD-1 sorted subsets. (B) Network analysis of significantly inferred genes and predicted targets. Node colors indicate fold change of gene expression between ex-vivo sorted CD160^+^PD-1^+^ and CD160^−^PD-1^+^ CD8 T cells sorted from 4 HIV viremic patients. The different shapes indicate genes in the different functional categories (see legend). (C) Histogram and scatter plot showing the MFI of KIR2DL2 and KIR2DL3 expression on DP and SP-PD-1 subsets in 6 HIV viremic patients.

Gene array profiling was performed on sorted CD8 T cell subsets based on CD160 and PD-1 co-expression in 4 HIV viremic individuals to determine if the functional defects observed in DP cells were the consequence of a distinct gene expression signature that was associated with signal transduction pathways that regulate T cell survival, turnover and function. Unsupervised cluster analysis showed that both DP and SP-PD-1 subsets clustered apart to create two statistically significant populations with unique transcriptional profiles (Figure 5A). The heatmap lists the top 39 genes expressed at significantly different levels between both subsets (*P*<0.05).

The results showed that genes upregulated in DP cells include those involved in the inhibition of several survival pathways. Importantly, SUMO2 (Small Ubiquitin-like modifier) was upregulated in DP CD8 T cells compared to SP-PD-1. This enzyme upregulates the activity of PIAS (protein inhibitor of activated STAT) molecules which are responsible for the inhibition of STATs (Signal Transducer and Activator of Transcription) including STAT5, a molecule directly downstream of γ chain cytokine receptors such as IL-7 and IL-15 [36]–[38]. The inhibition of the STAT5 pathway was confirmed by the downregulation of bcl-2 in DP cells (Figure 5B). Moreover DP cells upregulated the expression of KIF7, an antagonist of hedgehog the positive regulator of Wnt signaling [39] and several cell surface negative regulatory molecules including KIR2DL3 known to express ITIM motifs in the cytoplasmic tail [40]. In contrast, SP-PD-1 CD8 T cells upregulated the expression of several T cell activation markers including HAVCR2 (Tim-3), CTLA-4, LAT, CCR1 and TNFRS25 [26]–[27], [41]–[42]. In addition, positive regulators of Wnt/Notch signalling including Wnt7A and AXIN2 were upregulated in SP-PD-1 compared to DP CD8 T cells [43].

A network analysis of differentially expressed genes between DP and SP-PD-1 subsets showed a significant downregulation of signal transduction pathways enriched in genes that regulate T cell survival (IL-15, IL-7R, PIM3, bcl-2, all part of the STAT-5A pathway) and T cell effector function (LTβ, IL-18RAP, IL-18R1, CXCL16) in the DP subset (Figure 5B). Interestingly, the expression of NFκB was downregulated in DP compared to SP-PD-1 further confirming the advanced state of exhaustion and the unique identity of this novel subset in chronic HIV infection. Of note, TNFα production, which triggers NFκB [44], was downregulated in CD160^+^ CD8 T cells. Moreover, this network analysis confirmed the upregulation of several molecules with inhibitory functions in the DP subset such as the inhibitory KIR family (KIR2DL1, 2DL2, 2DL3, 2DL4, KIR3DL1, 3DL3) [40], 2B4 as well as members of the KLR family of proteins which are all associated to senescence. We confirmed by flow cytometry the increased expression of inhibitory KIR2DL2/KIR2DL3 in the DP compared to SP-PD-1 subset (p = 0.038) (Figure 5C). Taken together, gene expression analysis of CD160^+^PD-1^+^ CD8 T cells showed a gene expression signature that comprises several inhibitors of survival signal transduction pathways (STAT-5 and Wnt/Notch) and the increased expression of multiple immune inhibitory molecules. In contrast, SP-PD-1 CD8 T cells showed a transcriptional profile reminiscent of recently activated T cells.

## Discussion

The results presented here show that CD160 was upregulated on CD8 T cells during HIV infection. We identified 4 distinct subsets of CD8 T cells: DN, SP-PD-1, SP-CD160 and DP. Importantly, only CD8 lymphocytes that co-expressed CD160 and PD-1 had functional features and transcriptional profiles of exhausted cells. Recent studies have described an accumulation of inhibitory molecules on HIV-specific CD8 T cells. However, we show here that the distribution of cells expressing one or more of these molecules significantly shifted during the course of HIV infection. We show that SP-PD-1 cells increased in numbers in AHI while cells co-expressing CD160 and PD-1 were the dominant cell subset in CHI. This increased frequency of DP cells was associated with HIV disease progression and T cell dysfunction. Cells within the CD160^+^PD-1^+^ subset were less functional than SP-PD-1 and SP-CD160 as shown by the reduced frequency of cytokine secretion upon TCR triggering. Our gene array data confirmed the unique exhausted phenotype of the DP subset as these cells expressed transcriptional programs that were highlighted by the downregulation of the NFκB transcriptional node, strongly associated to T cell survival, and the upregulation of cell surface inhibitory KIR expression. This allowed us for the first time to provide molecular evidences for differences that demarcate cells expressing these inhibitory molecules as a consequence of T cell activation or those that express these molecules when they are functionally exhausted. We also show that CMV and HIV-specific CD8 T cell proliferation and cytokine secretion were rescued after blocking the engagement of CD160 with its natural ligand (HVEM). These results confirmed that functional exhaustion of T cells results from the progressive accumulation of several molecules that negatively impact on T cell activation. The temporal accumulation of these negative regulators results from chronic exposure to HIV and other molecules that trigger hyper-immune activation [45] since CD160 expression on T cells is observed mostly during the chronic phase of infection

Along with LIGHT, LTα, HSVgD, and BTLA, CD160 is also a ligand of HVEM. The interaction of LIGHT with HVEM delivers a co-stimulatory signal by triggering NFκB whereas the binding of CD160 or BTLA with this ligand delivers an inhibitory signal to CD4 T cells [35], [46], most probably by competing with LIGHT [47] for binding to HVEM. Although conflicting results regarding the function of CD160 have been reported [48]–[49], [50]–[51], recent findings are consistent with an inhibitory function for this molecule when expressed on T cells [20]–[21], [35]. The fact that SP-CD160 and SP-PD-1 subsets are expressed at comparable levels on HIV and CMV-specific T cells suggest that these cells are still functionally competent. Future work will compare the phenotype of HIV-specific CD8 T cells to other acute and chronic viral infections with the aim of understanding whether the observed phenotypic distribution is unique to HIV-specific CD8 T cells. Moreover since SP-PD-1 can still mount polyfunctional responses upon TCR triggering with cognate antigen, and the observation that HIV-specific T cells in ECs exhibit mostly an SP-CD160 phenotype further confirms the functionality of these subsets.

DP cells express the highest levels of PD-1 when compared to SP-PD-1.DP CD8 T cells are hence a unique dysfunctional T cell subset, as highlighted by the downregulation of several transcriptional nodes (STAT5, Notch-Wnt, NFκB) that regulate T cell survival and effector function as confirmed by flow cytometry and T cell functional assays. Differences in the functionality of DP and SP-PD-1 subsets could not be accounted for by their distribution in different memory or effector T cell compartments. Indeed our results (Figure S4) showed that DP and SP-PD-1 cells were found in T_TM_,T_EM_ and late differentiated T cells all known to be endowed mostly with effector functions thereby confirming the data by Yamamoto et *al.* [30]. These results are consistent with those generated in the LCMV model whereby the frequency of CD160^+^PD-1^+^CD8 T cells increases during CHI leading to an accumulation of dysfunctional HIV-specific CD8 T cells [20]–[21]. The expression of PD-1 in ST patients is significantly higher than that observed in ECs highlighting the ongoing viral replication in tissues (Figure S2). The higher levels of PD-1 on HIV-specific CD8 T cells from ST subjects most probably contribute to the dysfunction of these cells while DP cells from EC subjects still remain functional [17].

Previous studies have shown that CD160 and PD-1 interact with molecules downstream of the TCR [35], [52]. Following the activation of CD4 T cells with αCD3/CD28, ligation of CD160 with HVEM reduced the phosphorylation of tyrosine residues on several substrates such as CD3ζ. This decreased expression and phosphorylation of CD3ζ has been associated to T cell anergy and dysfunction [53]–[54]. Our results confirm that the presence of both CD160 and PD-1 on the surface of cells is required for the inhibition of TCR mediated signalling. In that context, we observed higher frequencies of functional antigen-specific CD8 T cells in lymphocytes negative for both CD160 and PD-1, whereas subsets that expressed either molecule alone were significantly less functional. It is important to note that both CD160 and PD-1 are upregulated upon T cell activation [55]. Hence it is more than likely that SP-PD-1 and SP-CD160 cells correspond to recently activated T cells that have upregulated those inhibitory receptors (PD-1, CD160) to control T cell activation as part of a homeostatic T cell response.

Blocking the interaction of CD160 and HVEM significantly enhanced CMV and HIV-specific proliferation and cytokine production further reinforcing the notion that CD160 acts as a negative regulator of T cell function. The magnitude of increase in CMV responses upon addition of αHVEM was more important than HIV-specific CD8 T cell responses (Figure 4). This is most probably due to the higher frequencies of DN and SP-CD160 cells within the CMV-specific T cell pool as compared to HIV-specific T cells. In addition, HIV-specific T cells include higher frequencies of DP cells compared to CMV-specific CD8 T cells. We found that the increase in proliferation that resulted from blocking CD160 engagement with HVEM was greater than that observed upon blocking PD-1 engagement with PD-L1 [24], [30]. As we have shown that CD160 is expressed at much higher levels than BTLA (the other ligand of HVEM), it is most likely that the rescue of HIV-specific CD8 T cell responses observed after addition of αHVEM targets mostly the interaction of HVEM with CD160. As previously shown, PD-1 is mostly expressed on DP cells during CHI. In CHI, the frequency of DP HIV-specific CD8 T cells is significantly higher than DP CMV-specific CD8 T cells (p<0.0001). HIV-specific DP cells are at an advanced stage of exhaustion and simultaneously express other negative regulatory molecules (elevated expression of KIR receptors), which could also contribute to T cell dysfunction. These cells, as shown from our transcriptional profiling, are hence truly exhausted and are at an irreversible stage of T cell dysfunction.

Several studies have observed that restoration of HIV-specific CD8 T cell proliferation and cytokine secretion of T cells specific for different epitopes [23]–[25] was variable. Our results suggest that the presence of multiple subsets of T cells expressing PD-1 with other negative regulators could account for this variability. For instance, in the murine LCMV infection model and during chronic HCV infection, subsets of PD-1^hi^ and PD-1^int^expressing cells have been identified. Blocking experiments have shown that only CD8 T cells that expressed intermediate levels of PD-1 were responsive to PD-1/PD-L1 blockade suggesting that not all specificities reverted towards a functional phenotype [56]–[57].

As noted above, PD-1 and CD160 are upregulated upon T cell activation. Transcriptional profiling helped elucidate the differences between cells that upregulate PD-1 as a result of T cell activation and PD-1^hi^ exhausted T cells. Indeed SP-PD-1 cells also expressed several other T cell activation markers (CTLA-4, Tim-3, CCR1, TNFRSF25) as well as other molecules associated with T cell survival (AXIN2, Wnt7A). In contrast, the KIR family of cell surface receptors clearly demarcated PD-1^hi^ (DP cells) exhausted cells from SP-PD-1 activated T cells [58]. Interestingly, only KIR genes with ITIM motifs (KIR2DL1, 2DL3, 2DL4, 3DL1, 3DL2, and 3DL3) were found upregulated on CD8 T cells which co-expressed CD160 and PD-1. The DP phenotype was associated with the down regulation of the NFκB survival pathway. As noted above, NFκB activity is triggered by LIGHT that competes with CD160 for binding to HVEM. These results support the view that DP lymphocytes express a large array of immune inhibitors and prevent CD8 T cells from acquiring a fully functional state. Our gene array results further confirmed the significant differences that demarcate DP from SP-PD-1 CD8 T cells. The former represent exhausted T cells while the latter are characterized by the expression of T cell activation markers and evidence for the induction of several pathways of T cell activation.

Our findings demonstrate that T cell exhaustion during chronic viral diseases results from the progressive temporal accumulation of multiple negative regulators of T cell activation and their interaction with their ligands. Understanding the contribution of these multiple inhibitory signals will be essential to properly define exhaustion and to determine whether these pathways converge to inhibit T cell activation by targeting multiple cellular pathways. In that context, system biology and transcriptional profiling have provided essential tools to dissect the functional status of cells expressing the different negative regulators of T cell activation. Functional restoration of exhausted T cell subsets will require combination therapies that target distinct sets of receptors at different stages of infection.

## Materials and Methods

### Ethics Statement

Written informed consent was provided by study participants and approved by the University of Montreal Health Center ethics review board (CRCHUM). Research conformed to ethical guidelines established by the ethics committee of the University of Montreal Health Center.

### Study Population

The study population included 38 HIV-1 subtype B infected individuals at various stages of infection and 7 HIV-uninfected donors (Table 1 and 2). HIV-infected patients were categorized into 4 subgroups: Elite controllers (ECs; n = 10) infected for more than 7 years with undetectable viremia, successfully treated (ST; n = 12) and aviremic individuals, subjects with acute HIV infection (AHI; n = 7) analyzed within 3 months of infection [59] and 9 chronically progressing subjects (CHI) infected for more than 6 months based on CD4 T cell counts under 500/mm^3^or declining CD4 T cell counts. All HIV infected subjects with the exception of ST, were naïve to antiretroviral therapy at the time of testing. Plasma viral loads were measured with the Amplicor HIV-1 Monitor Ultrasensitive method with a limit of detection of 50 HIV-1 RNA copies/mL of plasma (Roche Diagnostics, Mississauga, Canada). Absolute CD4 counts were quantified by the BD Multitest (CD3/CD4/CD8/CD45RA)using a FACSCanto (BD).

### Peptides and Tetramers

Soluble pMHC monomers were generated as previously described (Montreal, Canada) [60]. The peptides and tetramers used to analyze the CMV, EBV and HIV-specific CD8 T cell responses were: NLVPMVATV (A*02 CMV), TPRVTGGGAM (B*07 CMV), GLCTLVAML (A*02 EBV), RAKFKQLL (B*08 EBV), FLGKIWPSYK (A*02 Gag), ILKEPVHGV (A*02 Pol), SLYNTVATL (A*02 Gag), RLRPGGKKK (A*03 Gag), AIFQSSMTK (A*03 RT), QVPLRPMTYK (A*03 NEF), RYPLTFGWCF (A*23 NEF), RPGGKKKYKL (B*07 Gag), TPGPGVRYPL (B*07 NEF), SPAIFQSSM (B*07 RT), GEIYKRWII (B*08 Gag), FLKEKGGL (B*08 NEF), DCKTILKAL (B*08 Gag), RRWIQLGLQK (B*27 Gag) and YPLTFGWCF (B*35 NEF).

### Phenotypic Analysis

PBMCs were resuspended in PBS containing 2% FCS and stained with Tetramer-PE at 0.3 µg per 10^6^ cells. The following cocktail was used for phenotyping CD8 T cells: αCD160FITC (BD), αCD3Alexa 700 (BD), αCD8 PB (BD), αCD45RA ECD (Beckman Coulter), αCD27 APC-Cy7 (eBioscience), αCCR7 PE-Cy7 (BD), αCD158b PE (KIR2DL2, KIR2DL3) (BD) andαPD-1 APC (eBioscience). Monocytes, mDCs and pDCs were labelled using αCD3, αCD16 and αCD19 Alexa700 (BD), αCD14 PB (BD), αHLA-DR APC-Cy7, αCD11c APC, αCD123 PE (BD), αPD-L1 PE-Cy7 (BD) and αHVEM FITC (MBL). Dead cells were eliminated with an amine-reactive viability dye (LIVE/DEAD, Invitrogen). We acquired a minimum of 1×10^6^ events for all cytometry-related experiments using a BD LSRII flow cytometer and analyzed the results with FlowJo 9.1 (Treestar).

### Intracellular Cytokine Staining (ICS), CFSE and Multiplex Cytometric Bead Array (CBA)

Optimal peptides used for ICS and CFSE assays were identical to the ones folded in the pMHC monomers. PBMCs were stimulated with 5 ug/ml of CMV and HIV-specific peptides as described previously [24]. The cocktail used for ICS was: Tetramer PE, αCD160 FITC (BD), αTNFα Alexa 700 (BD), αIFN-γ PE-Cy7 (BD), αPD-1 APC, αCD3 PB and αCD8 ECD (Caltag). For CFSE, we stimulated PBMCs with CMV and HIV peptides for 6 days in the presence of 10 µg/ml of αPD-L1 (eBioscience), αHVEM (R&D systems)and polyclonal goat or monoclonal mouse IgG1 isotype controls (R&D systems).As described in the manufacturer's protocol, we used a cytokine bead array(CBA) (BD) assay to measure the concentrations of IL-4, IL-2, IL-10, TNFα and IFNγ in the supernatants harvested at the end of the CFSE assay.

### Gene Array Analysis

CD8 T cells subsets expressing CD160 and/or PD-1 were sorted from 4 HIV chronically infected individuals using BD FACS ARIA, lysed in RLT buffer and stored at −80°C. Total RNA was purified using RNA extraction kits (RNeasy Micro Kit, Qiagen).Quantification was performed using a spectrophotometer (NanoDrop Technologies) and RNA quality was assessed using the Experion automated electrophoresis system (Bio-Rad). Total RNA was amplified using the Illumina TotalPrep RNA Amplification kit [61]. Biotinylated cRNA was hybridized onto Illumina Human RefSeq-8 BeadChips and quantified using Illumina BeadStation 500GX scanner and Illumina BeadScan software.

Gene expression data was analyzed using Bioconductor(www.bioconductor.org) [62]. The R software package was used for pre-processing to filter out genes with intensities below background in all samples, minimum-replace (a surrogate-replacement policy) values below background using the mean background value of the built-in Illumina probe controls as an alternative to background subtraction, reduce “over inflated” expression ratios and finally quantile-normalize the gene intensities. Out of the 24526 initial probe set, 9070 probes were left after the filtering steps. The resulting matrix was log_2_ transformed and used as input for linear modeling using Bioconductor's *limma* package which estimates the fold-change between predefined groups by fitting a linear model and using an empirical Bayes method to moderate standard errors of the estimated log-fold changes for expression values from each gene [63]–[64]. *P* values from the resulting comparison were adjusted for multiple testing according to the method of Benjamini and Hochberg.

Gene networks were generated using Ingenuity Pathway Analysis (www.ingenuity.com). A dataset containing gene identifiers and corresponding statistical values were uploaded to the application. Each gene identifier was mapped to its corresponding gene in the Ingenuity Pathways Knowledge Base. Genes obtained from this analysis were overlaid onto a global molecular network. Networks of these focused genes were then algorithmically generated based on their connectivity.

### Statistical Analysis

Statistical analysis and graphical presentation was performed using GraphPad Prism 5.0c (GraphPad software, San Diego, CA) FlowJo 9.1 (Treestar) and SPICE 5.1 (http://exon.niaid.nih.gov) [65]. Two-tailed Unpaired *t*or Mann-Whitney tests were used to assess between-group differences (Figure 1). Two-tailed Wilcoxon matched pairs test was used to assess differences in the relative frequency of subsets over time (Figure 2), in the functionality between CD160 and PD-1 expressing subsets (Figure 3) and to assess differences in proliferative responses following co-culture with blocking antibodies (Figure 4). To determine if the variables analyzed came from a Gaussian distribution we applied the D'Agostino and Pearson's normality test. *P*-values less than 0.05 were considered statistically significant.

## Supporting Information

Figure S1 Heat map contrasting the differential genes expressed between CMV and HIV sorted tetramers in an HIV-infected cohort. Extracted mRNA for microarray gene transcription analysis was obtained by sorting HIV (top row: blue boxes) and CMV tetramers (top row: green boxes) from 27 HIV-infected subjects. This was done on 2 CMV and 11 HIV-specific tetramers. Each column in the heatmap represents a CMV or HIV specificity in the 27 HIV-infected subjects analyzed.
(TIF)

Figure S2 (A,C) Representative flow cytometry plots illustrating the expression of CD160 and PD-1 on total, CMV and HIV-specific CD8 T cells. PBMCs were labelled with fluorochrome conjugated αCD3, αCD8, αPD-1,αCD160 and HLA class I-matched tetramers (see materials and methods). Dying cells were eliminated with an amine-reactive viability dye (LIVE/DEAD). (B,D) Scatter plots represent the MFI and median frequencies of CD160 and PD-1 within CMV and HIV-specific CD8 T cells isolated from 7 HIV-1 uninfected and 38 HIV-infected subjects separated into four groups: 7 during acute infection (AHI), 9 chronic progressors (CHI), 12 successfully treated subjects (ST)and 10 Elite controllers (ECs). Blue and red dots represent CMV and HIV-specific CD8 T cells, respectively. *P*-values were determined by the unpaired *t* and Mann Whitney tests.
(TIF)

Figure S3 Median frequency and MFI of CD160 expression on total CD8 T cells from HIV-infected and uninfected individuals. PBMCs were labelled with fluorochrome conjugated αCD3, αCD8, αPD-1 andαCD160 (see materials and methods). Dying cells were eliminated with an amine-reactive viability dye (LIVE/DEAD). *P*-values were determined by the unpaired *t* and Mann Whitney tests.
(TIF)

Figure S4 Memory CD8 T cell distribution within CD160/PD-1 expressing subsets in HIV viremic individuals. PBMCs were labelled with fluorochrome conjugated αCD3, αCD8,αCD45RA, αCD27, αCCR7,αPD-1 andαCD160 (see materials and methods). Data was analyzed and presented using SPICE 5.1.
(TIF)

Figure S5 Median frequency of 4 distinct subsets of CD8 T cells: CD160^−^PD-1^−^ (DN), CD160^−^PD-1^+^ (SP-PD-1), CD160^+^PD-1^−^ (SP-CD160) and CD160^+^PD-1^+^ (DP)in HIV-infected and uninfected individuals. (A) Representative flow cytometry plots illustrating the co-expression of CD160 and PD-1 on CD8 T cells. (B) Scatter plots represent the average frequencies of CD160 and PD-1 expressing subsets within CD8 T cells isolated at different disease stages. (C) MFI of PD-1 expression on SP-PD-1 and DP CD8 T cells from HIV viremic subjects. PBMCs were labelled with fluorochrome conjugated αCD3, αCD8, αPD-1 andαCD160 (see materials and methods). Dying cells were eliminated with an amine-reactive viability dye (LIVE/DEAD). *P*-values were determined by Mann Whitney and unpaired *t* tests.
(TIF)

Figure S6 Longitudinal frequencies of HIV-specific CD8 T cells expressing PD-1 and CD160 during acute and chronic HIV infection. PBMCs were labelled with fluorochrome conjugated αCD3, αCD8, αPD-1,αCD160 and HLA class I-matched tetramers (see materials and methods). Dying cells were eliminated with an amine-reactive viability dye. Figures represent the total frequency of CD160 and PD-1 expression on HIV-specific CD8 T in 4 HIV-infected subjects. *P*-values were determined by the Wilcoxon matched pairs test.
(TIF)

Figure S7 Expression of HVEM on monocytes, mDCs and pDCs and PD-L1 on monocytes in HIV-infected and uninfected individuals. Monocytes, mDCs and pDCs were labelled using αCD3, αCD16, αCD19, αCD14, αHLA-DR, αCD11c, αCD123, αPD-L1 and αHVEM. Dead cells were eliminated with an amine-reactive viability dye (LIVE/DEAD). *P*-values were determined by the Mann Whitney *t* test.
(TIF)

Figure S8 Expression of CD160 and BTLA on total (B; n = 6) and HIV-specific CD8 T (C; n = 12) cells during chronic HIV infection. (A) Representative scatter plot of BTLA and CD160 co-expression on total and HIV-specific CD8 T cells.
(TIF)

Figure S9 Production of TNFα, IFNγ, IL-10, IL-4 and IL-2 following an HIV peptide-specific stimulation in the presence of isotype, αHVEM and/or αPD-L1 blocking antibodies. Supernatants harvested following a 6-day CFSE assay were used to assess cytokine production by cytokine bead array in the presence or absence of PD-1 and or CD160 engagement by their respective ligands. *P*-values were determined by a Paired *t* test and Wilcoxon matched pairs test.
(TIF)
