# Supplementary figures and images for: CD160 and PD-1 Co-Expression on HIV-Specific CD8 T Cells Defines a Subset with Advanced Dysfunction
Source: PLoS Pathog. 2012 Aug 16;8(8):e1002840. doi: 10.1371/journal.ppat.1002840 (PMC3420930; doi:10.1371/journal.ppat.1002840)

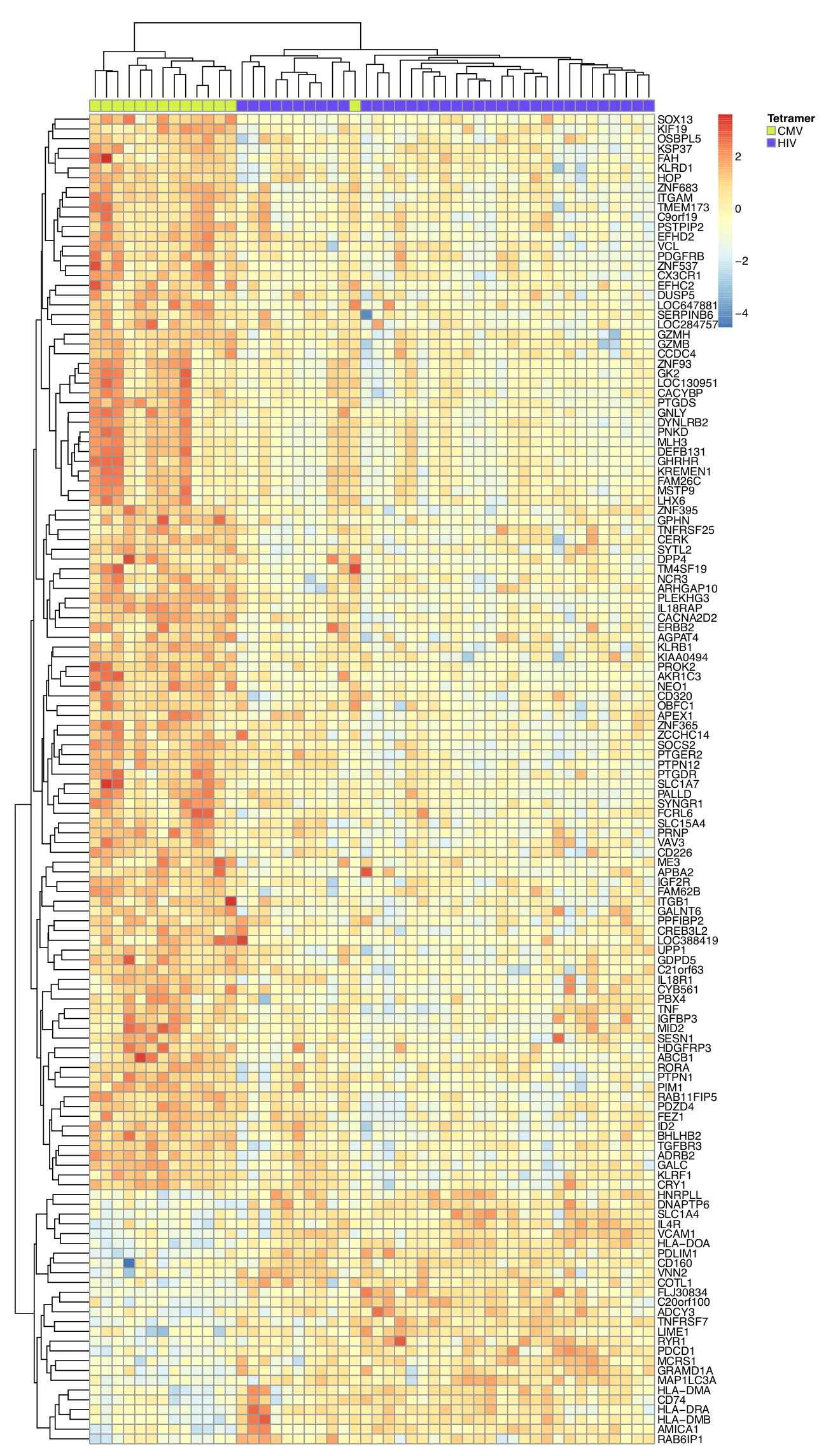

Supplement: Figure S1 — Heat map contrasting the differential genes expressed between CMV and HIV sorted tetramers in an HIV-infected cohort. Extracted mRNA for microarray gene transcription analysis was obtained by sorting HIV (top row: blue boxes) and CMV tetramers (top row: green boxes) from 27 HIV-infected subjects. This was done on 2 CMV and 11 HIV-specific tetramers. Each column in the heatmap represents a CMV or HIV specificity in the 27 HIV-infected subjects analyzed. (TIF) [file ppat.1002840.s001.tif]

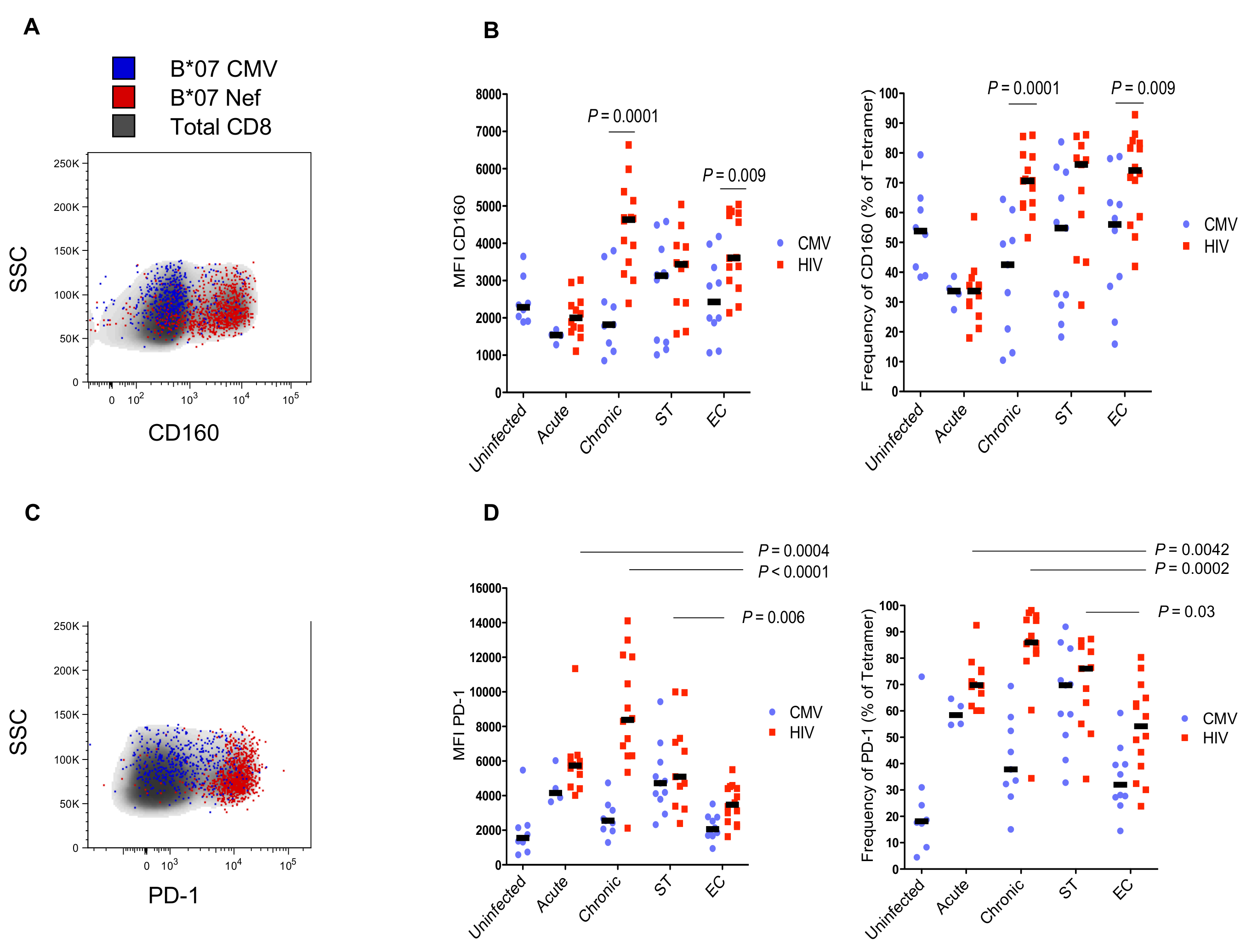

Supplement: Figure S2 — (A,C) Representative flow cytometry plots illustrating the expression of CD160 and PD-1 on total, CMV and HIV-specific CD8 T cells. PBMCs were labelled with fluorochrome conjugated αCD3, αCD8, αPD-1,αCD160 and HLA class I-matched tetramers (see materials and methods). Dying cells were eliminated with an amine-reactive viability dye (LIVE/DEAD). (B,D) Scatter plots represent the MFI and median frequencies of CD160 and PD-1 within CMV and HIV-specific CD8 T cells isolated from 7 HIV-1 uninfected and 38 HIV-infected subjects separated into four groups: 7 during acute infection (AHI), 9 chronic progressors (CHI), 12 successfully treated subjects (ST)and 10 Elite controllers (ECs). Blue and red dots represent CMV and HIV-specific CD8 T cells, respectively. P-values were determined by the unpaired t and Mann Whitney tests. (TIF) [file ppat.1002840.s002.tif]

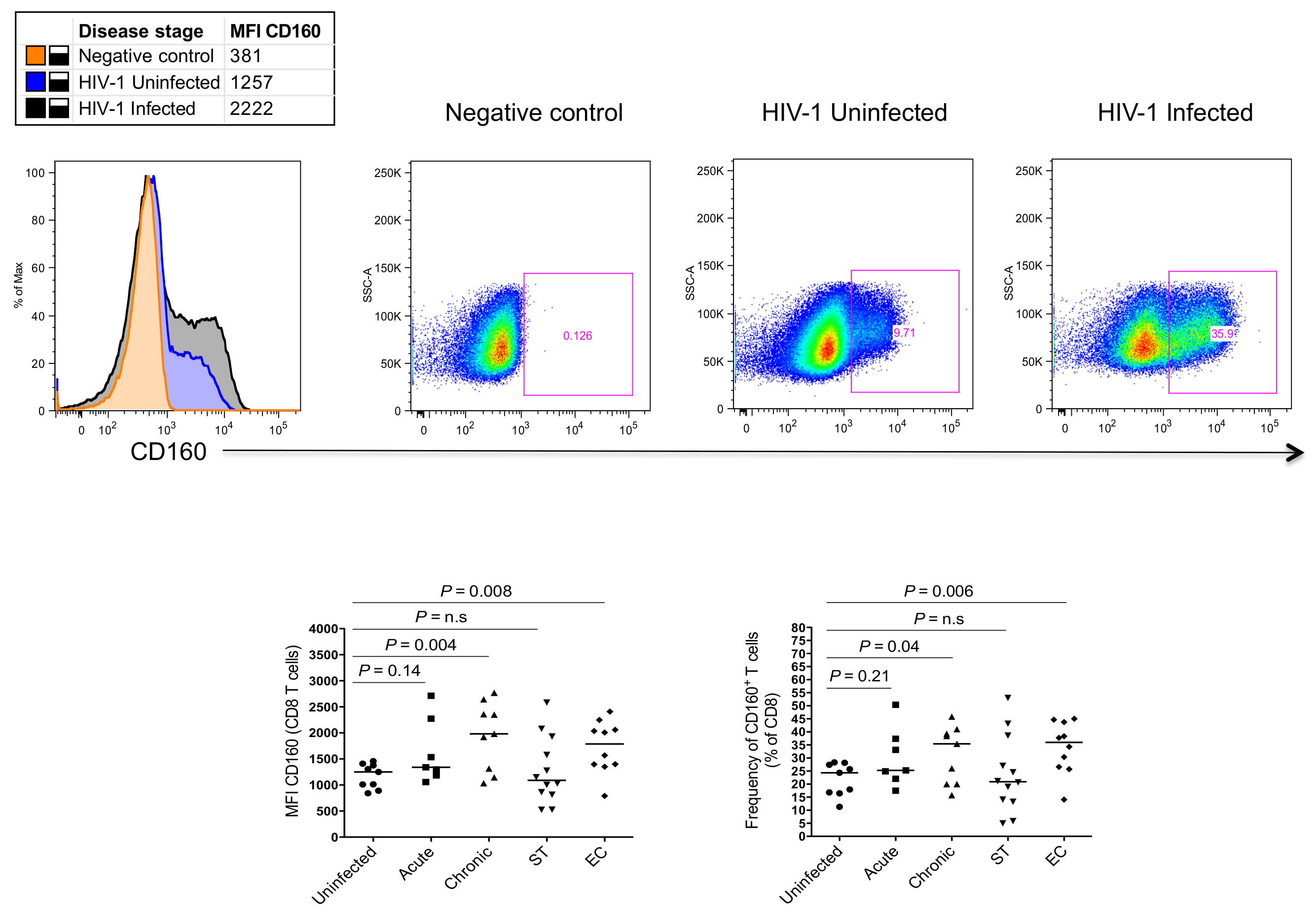

Supplement: Figure S3 — Median frequency and MFI of CD160 expression on total CD8 T cells from HIV-infected and uninfected individuals. PBMCs were labelled with fluorochrome conjugated αCD3, αCD8, αPD-1 andαCD160 (see materials and methods). Dying cells were eliminated with an amine-reactive viability dye (LIVE/DEAD). P-values were determined by the unpaired t and Mann Whitney tests. (TIF) [file ppat.1002840.s003.tif]

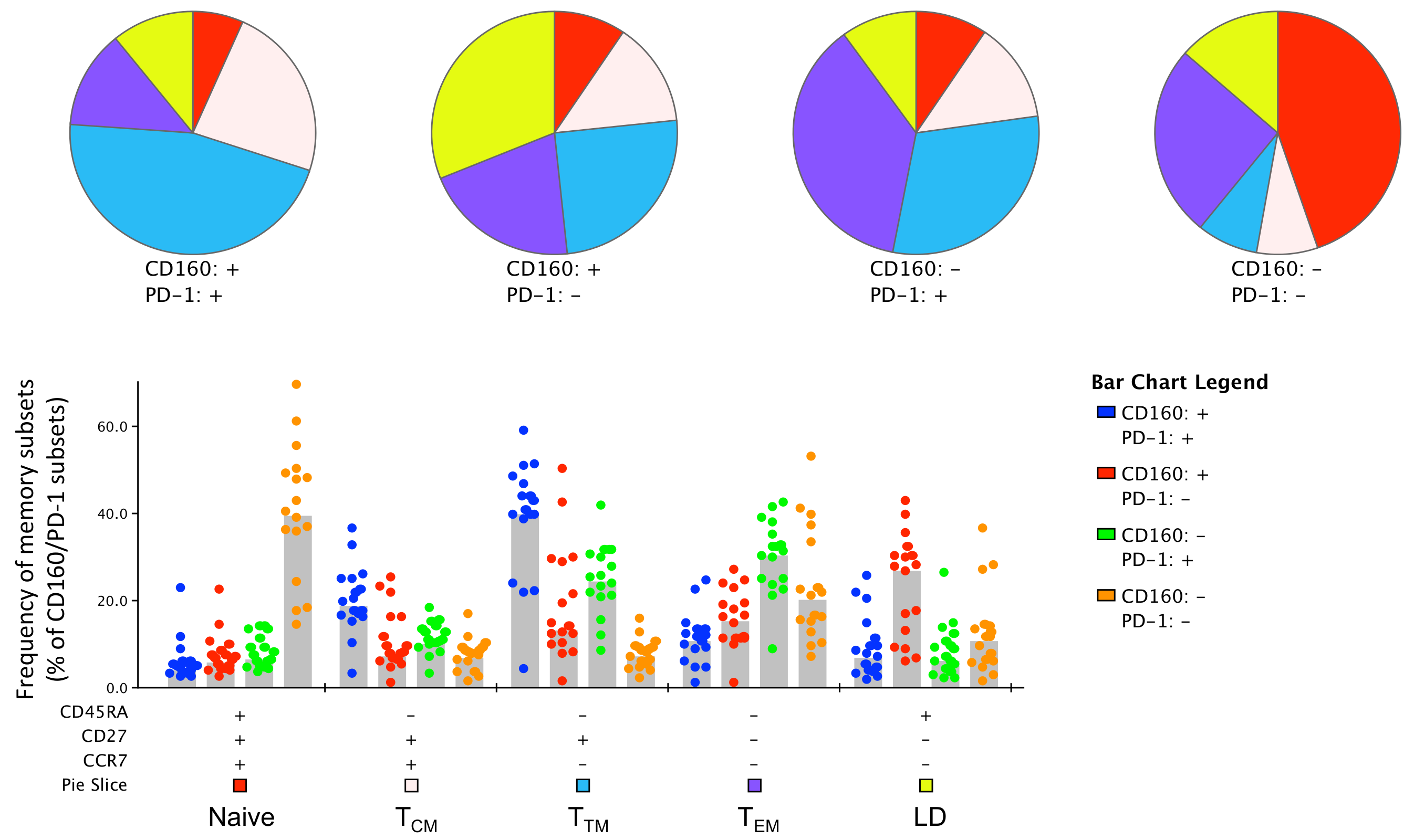

Supplement: Figure S4 — Memory CD8 T cell distribution within CD160/PD-1 expressing subsets in HIV viremic individuals. PBMCs were labelled with fluorochrome conjugated αCD3, αCD8,αCD45RA, αCD27, αCCR7,αPD-1 andαCD160 (see materials and methods). Data was analyzed and presented using SPICE 5.1. (TIF) [file ppat.1002840.s004.tif]

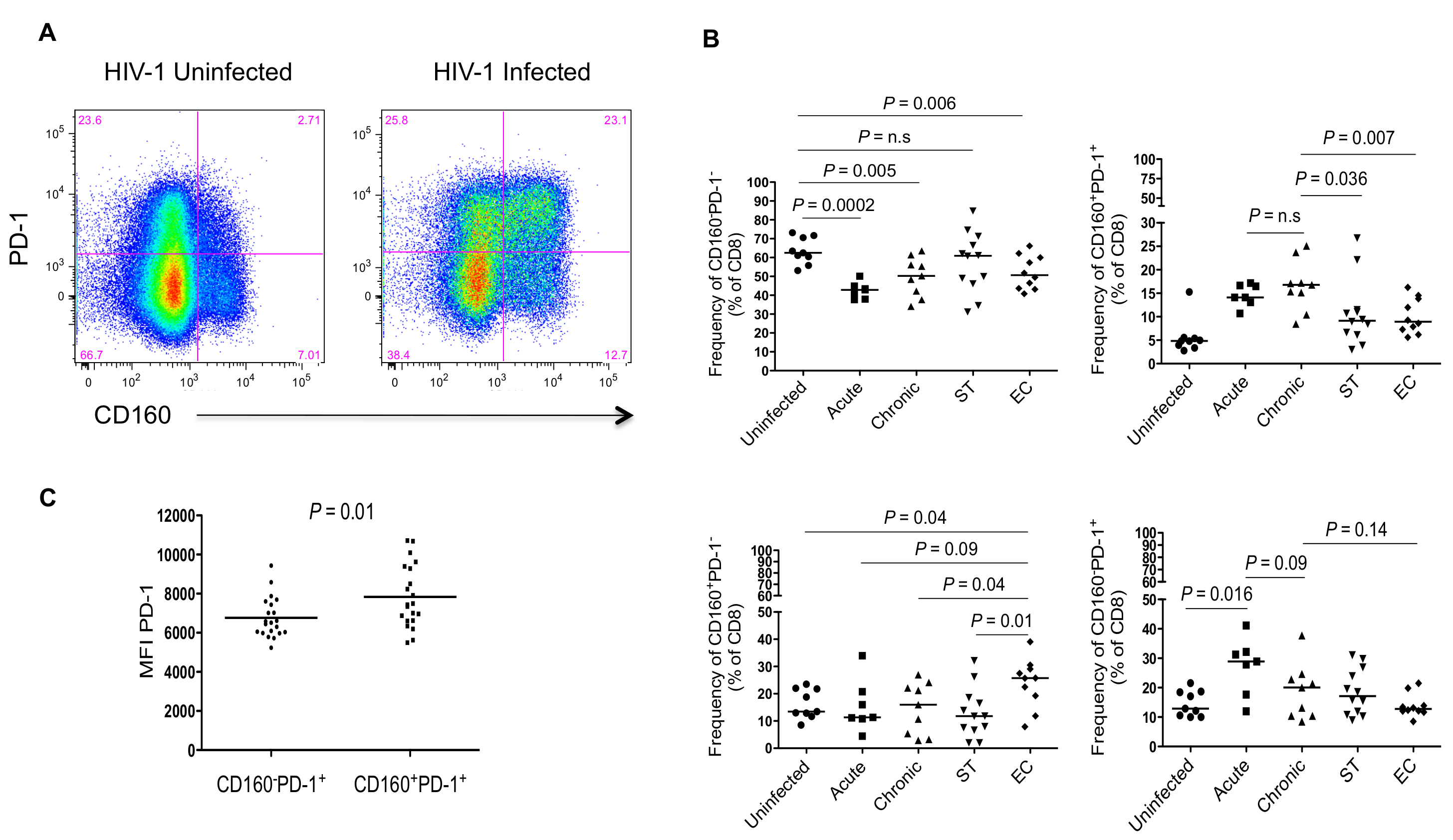

Supplement: Figure S5 — Median frequency of 4 distinct subsets of CD8 T cells: CD160−PD-1− (DN), CD160−PD-1+ (SP-PD-1), CD160+PD-1− (SP-CD160) and CD160+PD-1+ (DP)in HIV-infected and uninfected individuals. (A) Representative flow cytometry plots illustrating the co-expression of CD160 and PD-1 on CD8 T cells. (B) Scatter plots represent the average frequencies of CD160 and PD-1 expressing subsets within CD8 T cells isolated at different disease stages. (C) MFI of PD-1 expression on SP-PD-1 and DP CD8 T cells from HIV viremic subjects. PBMCs were labelled with fluorochrome conjugated αCD3, αCD8, αPD-1 andαCD160 (see materials and methods). Dying cells were eliminated with an amine-reactive viability dye (LIVE/DEAD). P-values were determined by Mann Whitney and unpaired t tests. (TIF) [file ppat.1002840.s005.tif]

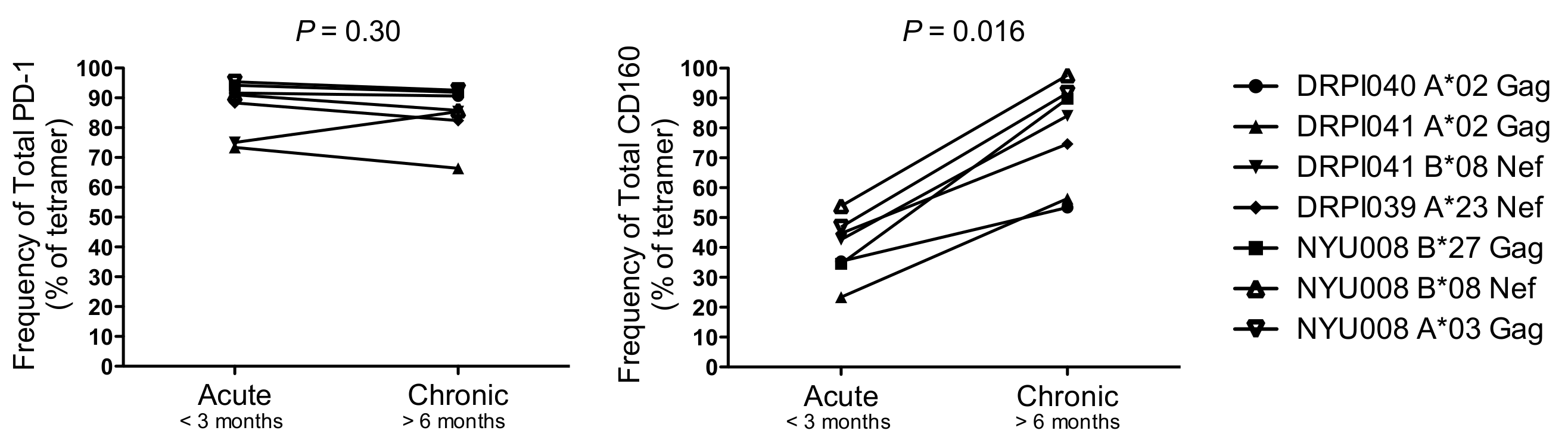

Supplement: Figure S6 — Longitudinal frequencies of HIV-specific CD8 T cells expressing PD-1 and CD160 during acute and chronic HIV infection. PBMCs were labelled with fluorochrome conjugated αCD3, αCD8, αPD-1,αCD160 and HLA class I-matched tetramers (see materials and methods). Dying cells were eliminated with an amine-reactive viability dye. Figures represent the total frequency of CD160 and PD-1 expression on HIV-specific CD8 T in 4 HIV-infected subjects. P-values were determined by the Wilcoxon matched pairs test. (TIF) [file ppat.1002840.s006.tif]

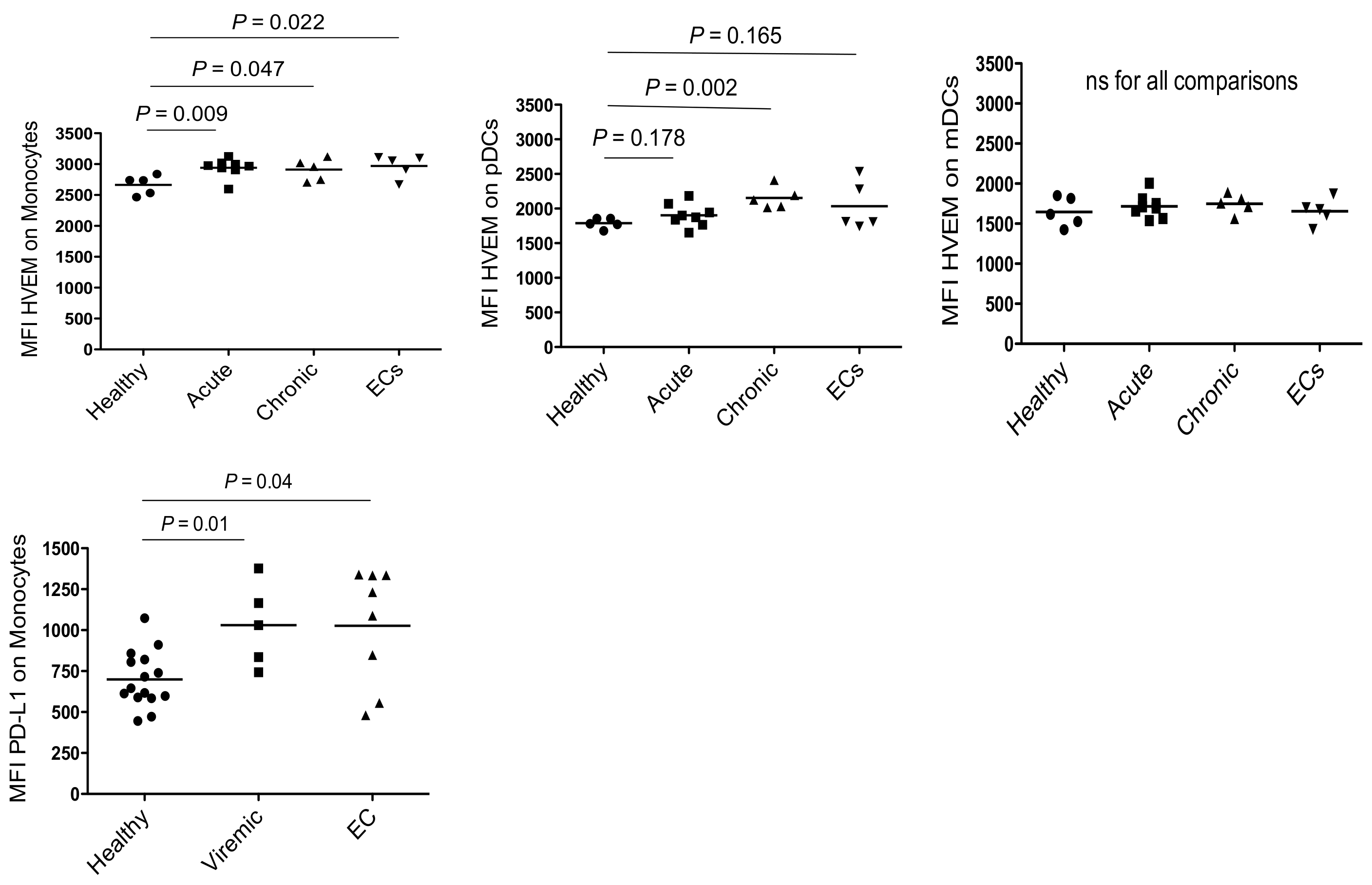

Supplement: Figure S7 — Expression of HVEM on monocytes, mDCs and pDCs and PD-L1 on monocytes in HIV-infected and uninfected individuals. Monocytes, mDCs and pDCs were labelled using αCD3, αCD16, αCD19, αCD14, αHLA-DR, αCD11c, αCD123, αPD-L1 and αHVEM. Dead cells were eliminated with an amine-reactive viability dye (LIVE/DEAD). P-values were determined by the Mann Whitney t test. (TIF) [file ppat.1002840.s007.tif]

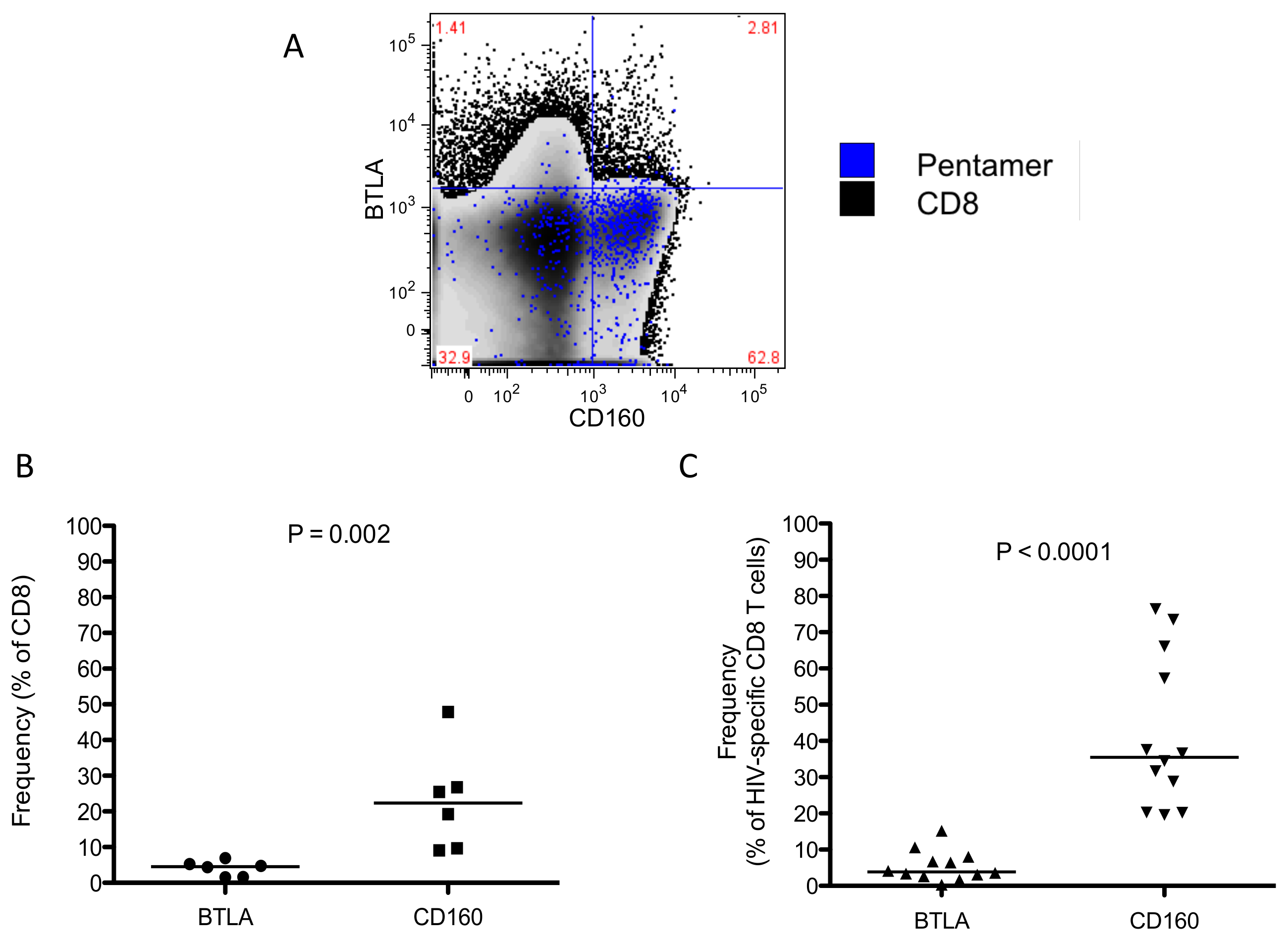

Supplement: Figure S8 — Expression of CD160 and BTLA on total (B; n = 6) and HIV-specific CD8 T (C; n = 12) cells during chronic HIV infection. (A) Representative scatter plot of BTLA and CD160 co-expression on total and HIV-specific CD8 T cells. (TIF) [file ppat.1002840.s008.tif]

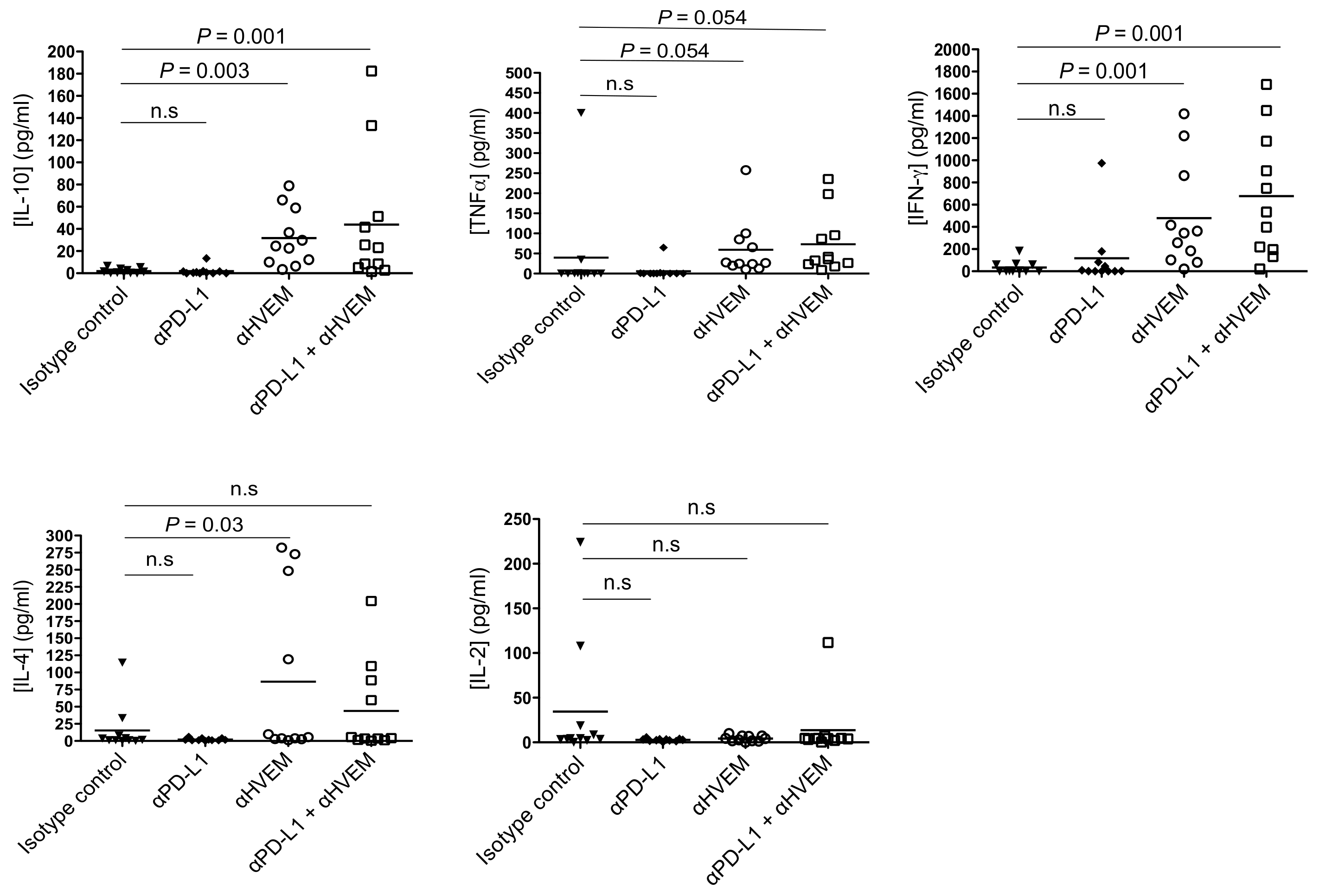

Supplement: Figure S9 — Production of TNFα, IFNγ, IL-10, IL-4 and IL-2 following an HIV peptide-specific stimulation in the presence of isotype, αHVEM and/or αPD-L1 blocking antibodies. Supernatants harvested following a 6-day CFSE assay were used to assess cytokine production by cytokine bead array in the presence or absence of PD-1 and or CD160 engagement by their respective ligands. P-values were determined by a Paired t test and Wilcoxon matched pairs test. (TIF) [file ppat.1002840.s009.tif]
